# Ecological, social, and intrinsic factors affecting wild orangutans’ curiosity, assessed using a field experiment

**DOI:** 10.1038/s41598-023-39214-2

**Published:** 2023-08-14

**Authors:** Caroline Schuppli, Lara Nellissen, Luz Carvajal, Alison M. Ashbury, Natalie Oliver-Caldwell, Tri Rahmaeti, Isabelle Laumer, Daniel Haun

**Affiliations:** 1 Development and Evolution of Cognition Research Group, Max Planck Institute of Animal Behavior, Bücklestrasse 5, 78467 Konstanz, Germany; 2 Leipzig Research Center for Early Child Development, Leipzig University, Jahnallee 59, 04109 Leipzig, Germany; 3 Department of Anthropology, University of Zürich, Winterthurerstrasse 190, 8057 Zürich, Switzerland; 4 Department of Éco-Anthropologie et Ethnobiologie, Muséum National d’Histoire Naturelle, CP 135, Rue Cuvier, 75 231 Paris Cedex 5, France; 5 Institute of Biology, Department of Comparative Cognition, University of Neuchâtel, Rue Emile-Argand 11, 2000 Neuchatel, Switzerland; 6 Department of Psychological and Brain Sciences, Johns Hopkins University, 3400 N. Charles Street, Baltimore, MD 21218 USA; 7 Department for the Ecology of Animal Societies, Max Planck Institute of Animal Behavior, Bücklestrasse 5, 78467 Konstanz, Germany; 8 Department of Biology, University of Konstanz, Universitätsstrasse 10, 78457 Konstanz, Germany; 9 Department of Biology, Graduate School, Universitas Nasional, Jalan Sawo Manila, RT.14/RW.3, Jakarta, 12550 Indonesia; 10 Department of Comparative Cultural Psychology, Max Planck Institute for Evolutionary Anthropology, Deutscher Platz 6, 04103 Leipzig, Germany

**Keywords:** Evolution, Psychology

## Abstract

The readiness to interact with and explore novel stimuli—i.e., curiosity—is the cornerstone of innovation. Great apes show broad and complex innovation repertoires. However, little is known about the factors that affect curiosity in wild apes. To shed light on wild apes’ curiosity, we measured the reactions of wild Sumatran orangutans (*Pongo abelii*) to an experiment apparatus. Overall, individuals were reluctant to touch the apparatus. However, compared to adults, immatures showed higher tendencies to explore (measured through looking durations and the probability of touching the apparatus) and to approach (measured through approach latencies and approach distances) the apparatus but were more likely to show behavioral signs of agitation. The presence of conspecifics who approached the apparatus increased visual exploration and approach tendencies. Prevailing habitat food availability positively affected visual exploration but had a negative effect on approach tendencies. These findings indicate that intrinsic, social, and ecological factors affect reactions to novelty in wild orangutans and suggest that exploration, neophobia and neophilia are independently regulated. Because reactions to novelty can be an essential pathway to innovation, our results suggest that factors acting on different elements of curiosity must be considered to understand the evolution of innovative tendencies.

## Introduction

Although novel objects and situations are rare in undisturbed natural habitats, where they do occur, they may afford important learning opportunities. Encountering and reacting to novel objects is, among others, one of the main pathways leading to innovations^1–6^. Individuals’ reactions to novel objects reflect their behavioral disposition^7^ and show how well they implement learning opportunities and how likely they are to make innovations^8–13^. Individuals who are more prone to interact with and explore novel stimuli, and thus are more likely to engage with learning opportunities, may develop adaptive skills and knowledge at a higher rate than more reluctant individuals. Skills and knowledge gained through innovation may increase an individual’s survival and reproduction by enabling the exploitation of a novel resource or the use of a current resource more efficiently^9,14–21^. Therefore, how well individuals realize learning opportunities may ultimately affect their fitness but see^22,23^, and see below.

Reacting to novel stimuli includes two major elements: the readiness to interact with the stimuli (determined by the interplay of one’s novelty responses) and the means used to investigate the stimuli (exploration). Novelty responses include *neophilia*, i.e., the spontaneous attraction to novel stimuli, and *neophobia*, i.e., the spontaneous aversion towards it^4,24^. *Exploration* describes the intensity and diversity of actions used to gather information about a stimulus through manipulation, visual examination, or other investigation^4,25,26^. Multiple lines of evidence from a range of taxa suggest that neophobia, neophilia and exploration are different mechanisms that are independently regulated and selected for^4,13,25,27–31^. This means that a single animal, given a single novel stimulus, can exhibit both a neophilic response (e.g., approaching the stimulus) and also a neophobic response (e.g., making threat vocalizations towards the stimulus) and may or may not, also explore the stimulus (e.g., visual exploration via focused starting, or manipulate the stimulus in various ways).

The interplay of novelty responses and exploration is used to study *curiosity* in animals, i.e., the motivation to know, learn and understand what is so far unknown^8,32–35^. Highly curious animals are more likely to exhibit neophilia and are more likely to explore novel stimuli than less curious animals (who are likely to, instead, exhibit neophobia). Whereas high levels of curiosity likely enhance the acquisition of fitness-relevant knowledge and skills, they also come with costs. Exploration is time intensive (which leads to opportunity costs) and potentially dangerous (as it reduces vigilance), just as high levels of neophilia bear a high risk of injury, poisoning, or predation^1,4,13,14,31,36,37^. These costs may outweigh the benefits of curiosity in most conditions and life stages. Accordingly, some studies have found negative effects of innovation ability on fitness e.g.^23^. Identifying the conditions which minimize the costs of exploration, neophilia or innovativeness in general will increase our understanding of how intrinsic curiosity can evolve. Ultimately, this may shed light on how selection can act on cognitive potential and thus on the evolution of cognition and innovation ability.

Investigating curiosity in non-human great apes (henceforth great apes) can lend particular insight to our understanding of the evolution of complex cognition, including human cognition, as they are humans’ closest relatives and have demonstrated high cognitive performance across different domains [e.g.^38–41^]. Furthermore, great apes show broad and complex innovation repertoires in the subsistence and comfort domains^42–44^. Several studies have looked at novelty responses and exploration in captive apes, but most focused on comparing species^7,45,46^. Only a few studies have investigated factors that affect within-species variation in curiosity: In chimpanzees and orangutans, social interactions and social cues lead to lower levels of neophobia and increased exploratory tendencies^46–48^. Whereas in orangutans, human contact and social housing positively affect individuals’ exploratory tendencies^8,49^, in chimpanzees, human demonstrators do not affect novelty responses^50^. In chimpanzees, exploratory tendency decreases with increasing age^51^.

Even less is known about great apes' novelty responses and exploration in socially and ecologically relevant contexts, i.e., in the wild. Studies that have compared wild and captive great apes’ responses to novelty revealed striking differences between the two settings^37,52^, suggesting that captive studies may have limited validity when it comes to understanding the underlying processes on the evolutionary level [see also results on other species, e.g.^35,53^]. Kalan and colleagues^54^ assessed novelty reactions of wild African great apes and found that chimpanzees showed stronger looking impulses towards camera traps (novel stimuli) compared to bonobos and gorillas. Furthermore, among these three species, immature individuals, compared to adults, and solitary individuals, compared to those with association partners, spent more time looking at the camera traps^54^, which is in line with the finding that juvenile chimpanzees are most likely to explore novel food items^55^. In terms of environmental factors, evidence from experiments with wild chimpanzees suggests that individuals are more likely to explore a novel foraging problem when they have a low, rather than high, energy balance^56^. Observational studies of wild great apes’ natural, everyday exploratory behavior show that immatures have the highest exploration rates^57,58^. Furthermore, wild orangutans’ exploration is socially induced on the developmental and proximate-immediate levels^59,60^, and more sociable populations tend to show higher exploration rates and more extensive innovation repertoires^59,61^.

These previous studies suggest that species, age, energy balance and social contexts can influence great apes’ curiosity in the wild. However, to fully comprehend the effects of these factors on novelty responses and exploratory tendencies, comprehensive testing of wild individuals is needed. Here, we aimed to experimentally test the impact of intrinsic and extrinsic factors on curiosity in wild orangutans. Specifically, we tested for differences between age-sex classes (intrinsic factor), the effects of association partners that approached the experiment apparatus (extrinsic social factor) and prevailing habitat food availability (extrinsic ecological factor). Orangutans are especially suitable for this question because of their semi-solitary lifestyle and slow development, which allows for testing individuals of different ages in different social settings. We developed a novel experiment apparatus and deployed it in the wild to test wild habituated orangutans’ reactions to novelty, including neophobia, neophilia, and exploration. We aimed to measure reactions to novelty in a naturalistic context, using naturalistic materials which resemble the types of novelty that individuals may encounter in their natural habitat. Based on past studies of curiosity in captive apes, novelty reactions in wild African apes, and naturally occurring exploration behavior in wild orangutans, we predicted that:

I) Immature orangutans show higher exploratory tendencies, levels of neophilia and lower levels of neophobia than adults.
II) The presence of association partners who themselves approach the apparatus reduces levels of neophobia and increases levels of neophilia and exploratory tendencies.
III) Low prevailing habitat food availability leads to increased exploratory tendencies, decreased neophobia and increased neophilia.

## Methods

### Data collection

We collected data at the Suaq Balimbing monitoring station in the Gunung Leuser National Park in South Aceh, Indonesia during two study periods: from June 2013 until March 2014 (first study period) and from February 2019 until March 2020 (second study period). If an individual participated in testing during both study periods, only trials from the first study period (i.e., the individual’s first testing period) were included in our analysis, as that is when we can assume that the test apparatus (henceforward called “experiment apparatus”) was novel to them and this avoids potentially confounding effects of a multi-year time gap between trials.

We conducted a total of 170 trials during individuals’ first testing periods, across 23 focal individuals, including 10 immatures (aged from 3 to 14 years), 5 mothers, and 8 unflanged males (i.e., adult males without secondary sexual characteristics). All focal individuals were already habituated to human observers, as part of long-term orangutan observation at the study site. Each individual participated in 1–27 trials (mean = 7.4 trials per individual). We excluded the data on immatures that were present during test trials but below the age of three years from the analysis, because they do not have the locomotor independence to approach the experiment apparatus on their own and are thus limited in how they can react to it. Furthermore, to ensure that we captured true novelty responses, the data on immatures that were present during the 2013–2014 study period and excluded because of their age, were also excluded from the 2019–2020 study period, even though they had by then reached the required age (N = 1).

**Figure 1:**
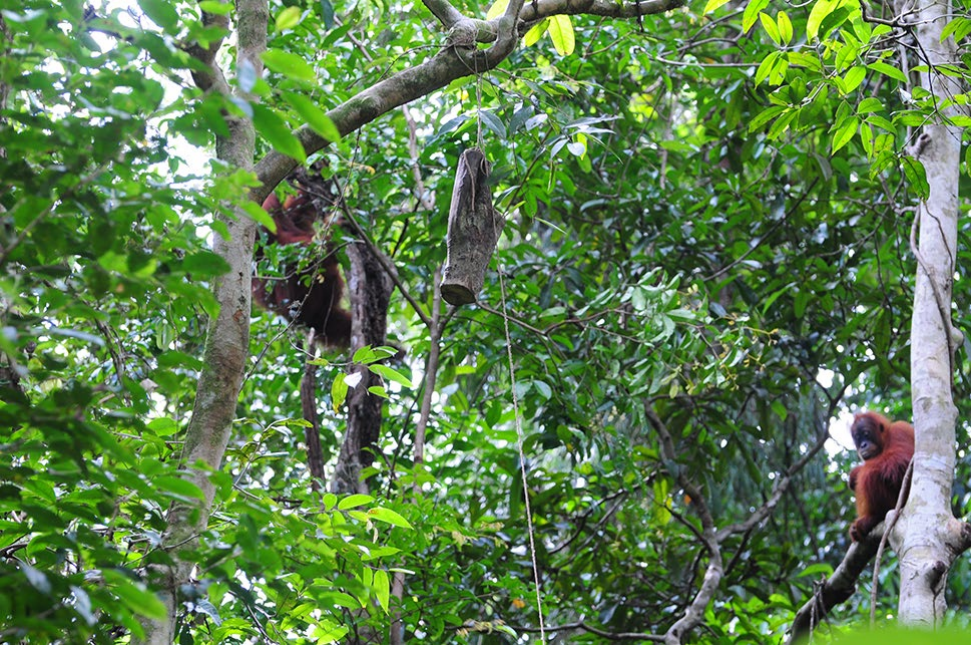
Experiment apparatus and set up. Experimental trial with the set-up experiment apparatus and two focal orangutans in the background.

The trials took place during full day focal animal follows. We waited to conduct the first trial until after the first extended feeding period of the day, which at Suaq usually happens within an hour after an orangutan leaves its night nest. The experiment apparatus (Fig. 1) was installed 5–20 m away from the focal animal while the focal animal was resting or feeding in a relaxed state. The initial distance between the focal orangutan and the experiment (exposure distance) was largely dependent on finding suitable spots to install the experiment apparatus in the canopy and was estimated by the observers (which were all trained in distance estimations as part of the ongoing behavioral data collection at the site, see www.aim.uzh.ch/de/orangutannetwork.html). Each trial began when the apparatus was installed in the canopy. To minimize the effect of the human observers on the focal individuals, we did not actively end trials by removing the apparatus when the focal individual was still in the vicinity of the experiment. Instead, trials ended when the focal individual retreated to more than 30 m from the apparatus (after which we never saw a subsequent approach). The average trial duration was 32 min (SD = 25, range = 6–145 min). Throughout the duration of these trials, when not directing their behavior towards the apparatus, the focal indiviudals were mostly resting or feeding. Furthermore, on days when the experiment was performed with multiple individuals (see below), there were never any signs of agitation or agonism between these indiviudals. Because we find and follow the orangutans opportunistically, we could not conduct the experiments on a predetermined timeline, and experimental trials were conducted opportunistically as well. However, to control for their potential confounding effects, we control for the number of previous trials and the exposure distance in our statistical models (see below).

Previous studies have shown that wild orangutans are highly neophobic and hardly ever approach objects that are made of foreign materials^52^. Therefore, in contrast to most classic novelty studies, we chose natural material for our experiment apparatus and a familiar food reward: The experiment apparatus was a *ca.* 65cm-long wooden log with a natural tree hole, which we filled with locally harvested forest honey. We additionally attached honeycombs to the outside of the apparatus, to increase the focal individuals’ interest in the apparatus. The apparatus was hung from a branch in the canopy using a green plastic rope. To install the apparatus, we shot a stone, attached to a fishing line that was attached to the green rope, up over a tree branch using a hand-held wooden slingshot; the fishing line, and then green rope, were then pulled over the branch until the apparatus was well up into the canopy (10–20 m high). Once the apparatus was secured in place, the observers retreated several meters to make sure that nobody stood directly below the experiment apparatus.

Because the trials were conducted in the trees, visibility was sometimes restricted. We therefore had three separate observers collecting data during the trials whenever possible: One observer filmed the focal animal, a second observer watched and narrated what they saw (which was recorded on the same or an additional camera that was used by the first observer, depending on the distance between the filming and narrating observer), and a third observer noted all measured parameters on a data sheet. All measured parameters were later obtained directly from the video, and missing elements were filled-in using the narration and/or, when needed, the data sheet.

### Variables

To quantify individuals’ reactions to the apparatus, we measured 6 parameters, which served as response variables in our statistical models:

A) *Looking duration: as a measure of visual exploration*^30,54,62^**,** measured as the number of seconds a focal was looking at the experiment apparatus during a trial.
B) *Approach latency: as a measure of neophilia*^6,8^**,** measured as the time between when the experiment apparatus was in place and when the focal individual started to approach it.
C) *Approach distance: as a measure of neophilia*^52,63^**,** measured as the distance over which the focal individual moved to approach the experiment apparatus which was calculated by subtracting the closest distance from the initial exposure distance. By including the exposure distance as a control variable in our statistical model, we analyzed the distance change relative to the exposure distance (see below).
D) *Agitation: as measure of neophobia*^4,8,64^, measured by the presence of behavioral indicators of agitation, namely scratching ^65,66^, as well as “kiss squeak,” “grumble”, “grunt”, “crying”, and “grumph” vocalizations^67^; see aim.uzh.ch/de/orangutannetwork/orangutancallrepertoires.html for details on these vocalizations.
E) *Touch: as a measure of exploration*^68,69^**,** measured by whether the focal individuals touched or did not touch the apparatus (or the honeycombs, via direct body contact or by using tools).
F) *Latency to leave the vicinity of the experiment: as a measure of neophobia*^54,63^**,** measured as the time between the start of the experiment and when the focal individual retreated to a distance of 30 m from the experiment apparatus.

To determine the factors that affected individuals’ reactions to the apparatus, we investigated:

1. the focal individuals’ *age-sex class* (divided into immatures, mothers, and unflanged males),
2. the *presence of an approaching party member* (i.e., at least one association partner who decreased its distance to the apparatus during the trial, whereby orangutans of all age-sex classes within 50 m of the focal individual were considered as party members), and
3. the *current habitat food availability*. Food availability was quantified monthly via the percentage of trees bearing fruit in an established phenology plot in the study area which consisted of approximately 1000 marked trees (see^70^ for details on the food availability data collection method).

We included these three factors as predictor variables in our statistical models (see below).

To control for possible confounding external effects that could not be held constant, we included the initial exposure distance (i.e., the distance between the focal individual and the experiment apparatus when it was set up), and the trial number (i.e., the individual-specific order of trials) as fixed control effects.

When multiple individuals were present during the experiment, data was collected on all of them (i.e., they were all considered as focal individuals in their own experimental trial and as association partners in the others’ experimental trials at the same time).

### Statistical analyses

We analyzed and visualized the data using the R programming language^71^, and RStudio^72^. To investigate the effects of the independent variables on the four dependent variables, we used linear mixed effect regression models (LMER) with a Gaussian family distribution (for the models with continuous response variables, i.e., looking duration, approach latencies, and approach distances) and a generalized linear mixed model (GLMM) with a Binomial family distribution (for the model with a binary response variable, i.e. the presence of signs of agitation), as implemented in the lme4 package^73^. Upon visual inspection of the distribution of each continuous response variable, we log transformed all three; looking duration (log(looking duration + 1)), approach latency (log(approach latency + 1), and approach distance (log(distance change + 1)). We explored the most suitable random effect structure for our models using model selection via likelihood ratio tests with the *anova* function^74,75^. Model selection indicated that we should use models with no random intercepts or random slopes. However, most focal individuals contributed to multiple data points (i.e., participated in multiple trials, see above) and thus, to avoid pseudo replication issues, we included the focal individual as a random intercept in all our statistical models. Following this procedure, all models converged, and none had singularity issues.

For each of the six models, we first tested the overall fit of the model by comparing the full model (including all predictor variables and random effect) with the null model (including only random effect and control variables) using a likelihood ratio test with the *anova* function^74,75^. For the full models that were supported, we assessed the significance of the predictor variables via their *p-*values in the full model (in the case of the GLMM with a Gaussian family distribution, the *p-*values were computed with the *cf-test* function from the multcomp package^76^). We investigated differences between the age-sex classes using posthoc tests as implemented in the *glht* function of the multcomp package^76^.

We visually examined all model fits to assess whether they satisfied model assumptions (for the LMERs this included normally distributed model residuals, homogeneity of the variance, and normally distributed random effects) and to check for the presence of influential observations^77^. For the Binomial GLMMs, we tested for overdispersion and zero inflation as implemented in the DHARMa package^78^. We checked all models for multicollinearity with the *check_collinearity* function of the performance package^65^. We did not find any evidence for multicollinearity issues in our models (variance inflation factors ranged from 1.01 to 1.21 across the models and factors).

To assess the overall goodness-of-fit of the models, we used model R^2^ values, which we retrieved via the r2glmm package^79^ following^80^. We assessed the stability of all our mixed models on the level of the random effects by dropping levels one-by-one. We found that the direction of the effects of the predictor variables on the response variables were consistent across model recomputations.

For the plots, we used the ggplot2 and cowplot packages^81,82^, and calculated the marginal effects of each predictor variable (while holding all other variables at their means) from each corresponding model using the ggeffects package^83^. For plots based on models with log transformed response variables, we back transformed the predictions so that the y-axes are on the original scale of the measured variable rather than on the log scale.

### Ethics statement

All our research protocols were approved by the Ministry of Research, Technology and Higher Education (RISTEKDIKTI; Research Permit No.: 54/E5/E5.4/SIP/ 2019 and following) and adhered to the legal requirements of Indonesia.

## Results

### General model fits

**Figure 2:**
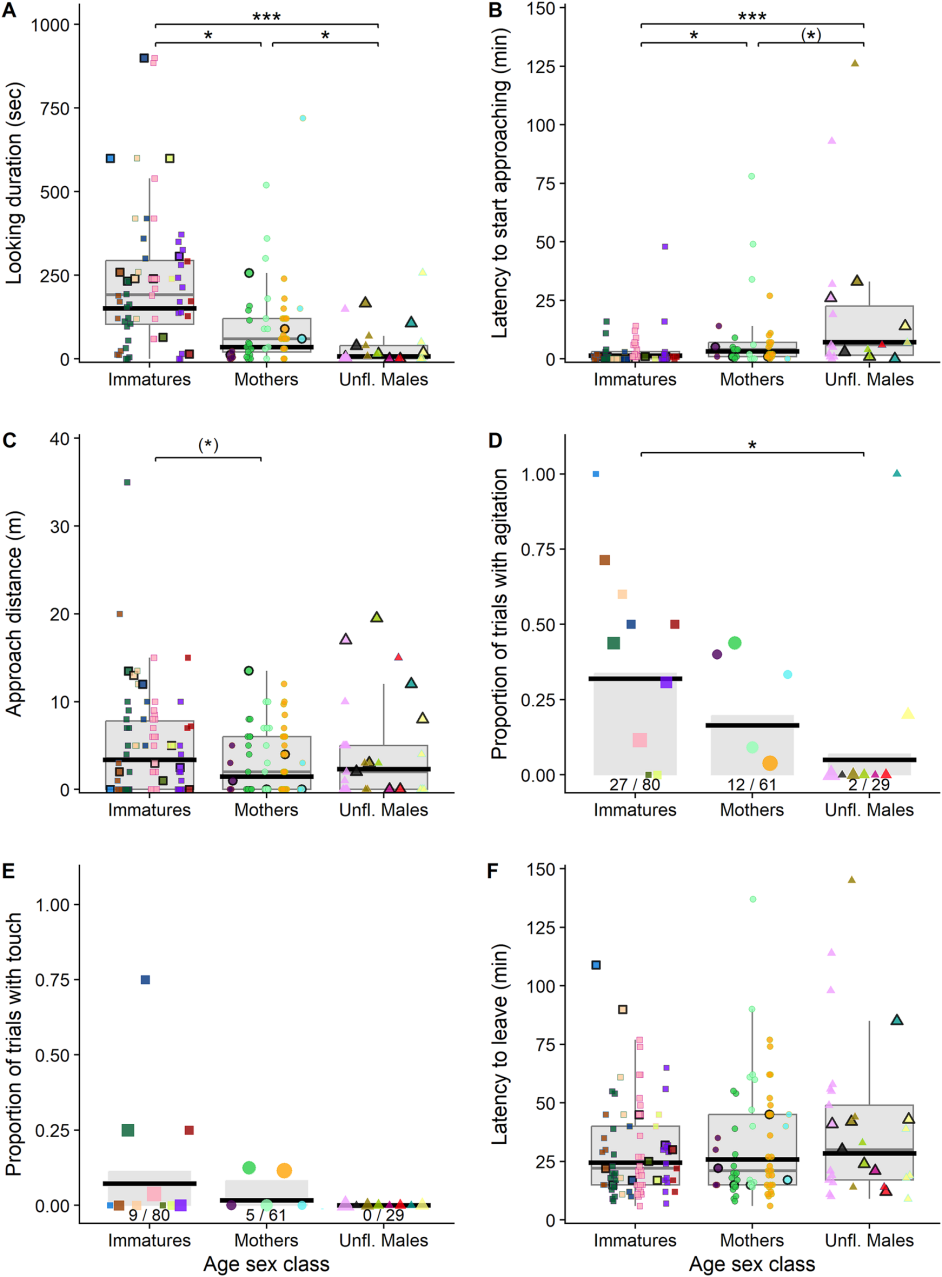
Differences between age-sex classes in reactions to the experiment apparatus. (**A**) Looking durations at the experiment apparatus, (**B**) latencies to approach the experiment apparatus, (**C**) approach distances towards the experiment apparatus, (**D**) the probability of signs of agitation during the experimental trial, (**E**) the probability of touching the apparatus, and (**F**) the latency to leave the vicinity of the experiment apparatus for immatures, mothers and unflanged males. For panels (**A**–**C**) and (**E**), each data point represents one experimental trial on one focal individual with colors referring to different individuals. The grey boxes show the interquartile ranges, the whiskers extend to the maximum and minimum data points that are within 1.5 times the interquartile range from the upper and lower quartiles, and the grey horizonal lines represent the medians. For panels (**D**) and (**F**), the grey columns represent the average proportion of trials with agitation/touch for each age-sex class (with the number of trials with agitation/touch over the total number of trials per age-sex class indicated under each grey bar) and the data points each show one individual’s proportion of trials with agitation; the size of each data point represents the number of trials for that individual. In all panels, the thick black horizontal lines represent mean model predictions when holding all other predictor variables at their means and the data points with black borders depict the first trial of each individual.

**Figure 3:**
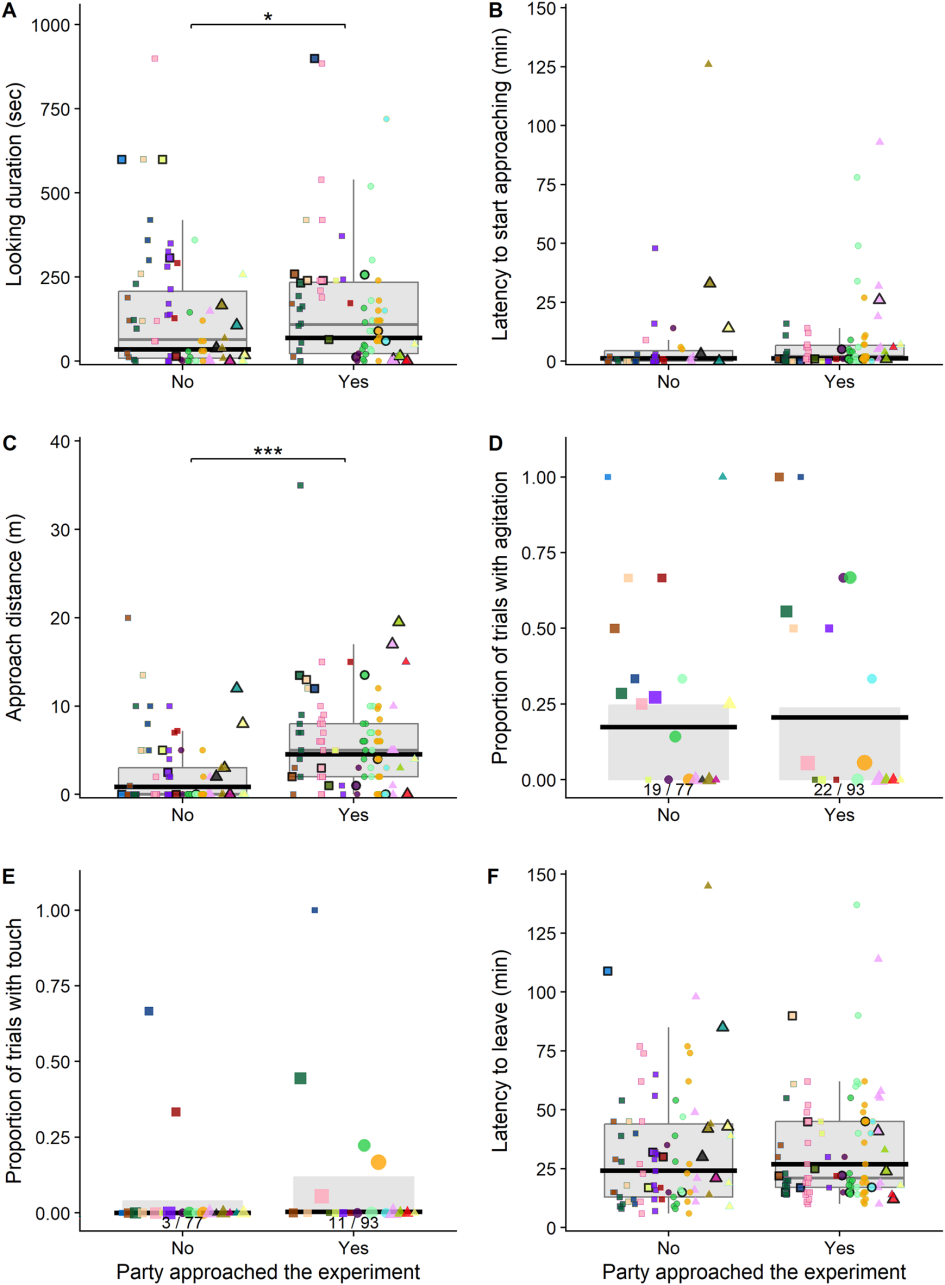
Effects of association partners on reactions to the experiment apparatus. (**A**) Looking durations at the experiment apparatus, (**B**) latencies to approach the experiment apparatus, (**C**) approach distances towards the experiment apparatus, (**D**) the probability of signs of agitation during the experimental trial, (**E**) the probability of touching the apparatus, and (**F**) the latency to leave the vicinity of the experiment apparatus when there was no association partner (party) approaching the experiment apparatus versus when there was. For panels (**A**–**C**) and (**F**), each data point represents one experimental trial on one focal individual with colors referring to different individuals. The grey boxes show the interquartile ranges, the whiskers extend to the maximum and minimum data points that are within 1.5 times the interquartile range from the upper and lower quartiles, and the grey horizonal lines represent the medians. For panels D and E, the grey columns represent the average proportion of trials with agitation/touch for each age-sex class (with the number of trials with agitation/touch over the total number of trials per age-sex class indicated under each grey bar) and the data points each show one individual’s proportion of trials with agitation; the size of each data point represents the number of trials for that individual. In all panels, the thick black horizontal lines represent mean model predictions when holding all other predictor variables at their means and the data points with black borders depict the first trial of each individual.

**Figure 4:**
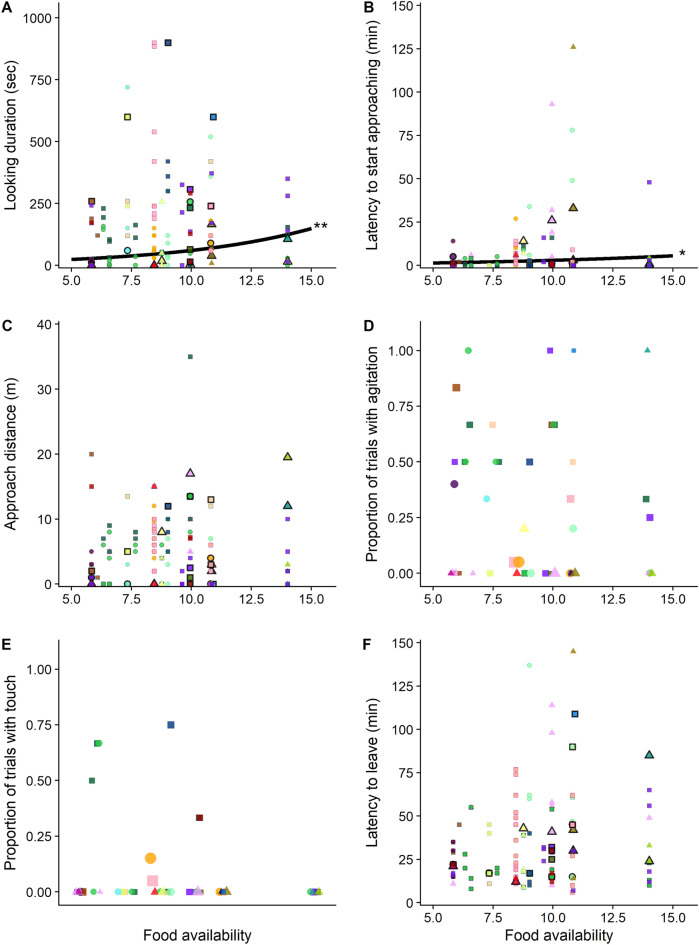
Effects of food availability on reactions to the experiment apparatus. (**A**) Looking durations at the experiment apparatus, (**B**) latencies to approach the experiment apparatus, (**C**) approach distances towards experiment apparatus, (**D**) the probability of signs of agitation during the experimental trial, (**E**) the probability of touching the apparatus, and (**F**) the latency to leave the vicinity of the experiment apparatus as a function of habitat food availability. For panels (**A**–**C**) and (**F**), each data point represents one experimental trial on one focal individual with colors referring to different individuals, and shape showing their age-sex class (square = immature, circle = mother, triangle = unflanged male). The thick black lines represent mean model predictions across food availability values for significant effects, when holding all other predictor variables at their means and the data points with black borders depict the first trial of each individual. For panels D and E, the data points each show one individual’s proportion of trials at that food availability value with agitation/touch; the size of each data point represents the number of trials for that individual at that food availability value. Note that points in panels D and E have been slightly jittered horizontally, to ensure that all points are visible.

Model comparison showed that five of the six full models fitted the data better than their respective null models, indicating an overall effect of the predictor variables on the response variables (LRTs full model versus null model: looking duration (model A): Chi-square = 26.842, *P *< 0.001; approach latency (model B): Chi-square = 19.396, *P *< 0.001, distance change (model C): Chi-square = 52.506, *P *< 0.001; agitation (model D): Chi-square = 9.397, *P *= 0.052; touch: Chi-square = 9.184, *P *= 0.057 (model E). For the model on the time to leave the vicinity of the experiment the full model did not fit the data better than the null model: Chi-square = 4.393, *P *= 0.355 (model F). Therefore, we did not analyze model F in more detail (but see Figs. 2F, 3F, and 4F for the according plots).

### Differences between age-sex classes in reactions to experiment apparatus

We found that immature individuals looked significantly longer at the experiment apparatus than mothers and unflanged males, and that mothers looked significantly longer at the apparatus than unflanged males (Table 1—model A, Fig. 2A; the mean looking duration of immatures was 233 s, of mothers 103 s, and of unflanged males 34 s). There was no statistically significant difference between the age-sex classes in the probability to touch the experiment apparatus (Table 1 model E). However, four immatures and two mothers touched the apparatus in a total of 14 trials (see discussion for more details Fig. 2E), whereas no unflanged male ever touched the apparatus. Immature individuals had significantly shorter latencies to approach the experiment apparatus than mothers and unflanged males and there was a trend for mothers to approach the apparatus faster than unflanged males (Table 1—model B, Fig. 2B; the mean approach latency of immatures was 3.4 min, of mothers 8.6 min and of unflanged males 19.9 min). There was a trend for immatures to approach closer to the experiment apparatus (measured by distance change towards the apparatus and controlled for initial exposure distance) than mothers but no evidence for differences in approach distances between the other age-sex classes (Table 1—model C, Fig. 2C). Immatures also had a higher probability of showing signs of agitation during the experimental trials compared to unflanged males, while there were no differences in agitation probability between the other age-sex classes (Table 1—model D, Fig. 2D).

**Table 1:** Effects of age-sex class of the focal individual, habitat food availability, the presence of party members who approached the apparatus on A) looking duration, B) approach latency, C) approach distance and D) probability of agitation (i.e., the presence of signs of agitation), controlled for the initial exposure distance, trial number (i.e., the individual-specific trial order).

Model	Response	Factors	Factor Type	Estimate	Std. Error	*P*-Value
A	Looking duration	Intercept	Intercept	3.769	0.807	< 0.001
		Age-sex Class	Predictor			
		Immatures—Mothers		− 1.444	0.595	**0.031**
		Immatures—Unfl. Males		− 2.902	0.602	< **0.001**
		Mothers—Unfl. Males		− 1.457	0.679	**0.032**
		Food availability	Predictor	0.178	0.068	**0.009**
		Party approached	Predictor	0.653	0.294	**0.027**
		Exposure distance	Control	− 0.052	0.032	0.107
		Trial number	Control	− 0.014	0.026	0.573
		Individual	Random			
B	Approach Latency	Intercept	Intercept	− 0.775	0.562	0.168
		Age-sex Class	Predictor			
		Immatures—Mothers		0.580	0.253	**0.044**
		Immatures—Unfl. Males		1.230	0.300	< **0.001**
		Mothers—Unfl. Males		0.650	0.337	0.054
		Food availability	Predictor	0.100	0.051	**0.049**
		Party approached	Predictor	0.013	0.248	0.957
		Exposure distance	Control	0.033	0.023	0.154
		Trial number	Control	0.047	0.017	0.006
		Individual	Random			
C	Approach distance	Intercept	Intercept	0.143	0.387	0.711
		Age-sex Class	Predictor			
		Immatures—Mothers		− 0.572	0.243	0.056
		Immatures—Unfl. Males		− 0.277	0.262	0.581
		Mothers—Unfl. Males		0.295	0.290	0.581
		Food availability	Predictor	0.017	0.033	0.611
		Party approached	Predictor	1.096	0.139	< **0.001**
		Exposure distance	Control	0.047	0.015	0.002
		Trial number	Control	0.006	0.012	0.623
		Individual	Random			
D	Agitation	Intercept	Intercept	2.167	1.182	0.067
		Age-sex Class	Predictor			
		Immatures—Mothers		− 0.840	0.619	0.321
		Immatures—Unfl. Males		− 2.189	0.895	**0.043**
		Mothers—Unfl. Males		− 1.349	0.962	0.321
		Food availability	Predictor	− 0.121	0.105	0.232
		Party approached	Predictor	0.211	0.434	0.628
		Exposure distance	Control	− 0.087	0.049	0.077
		Trial number	Control	− 0.116	0.043	0.007
		Individual	Random			
E	Touch	Intercept	Intercept	0.662	2.524	0.793
		Age-sex Class	Predictor			
		Immatures—Mothers		− 1511	1.605	1
		Immatures—Unfl. Males		− 18.927	4954	1
		Mothers—Unfl. Males		− 17.416	4954	1
		Food availability	Predictor	− 0.268	0.212	0.207
		Party approached	Predictor	1.449	0.918	0.114
		Exposure distance	Control	− 0.196	0.118	0.096
		Trial number	Control	0.082	0.059	0.169
		Individual	Random			

Including estimate, standard errors, and *p*-values, analyzed with GLMMs with a Gaussian (models A, B, C) or Binomial (models D, E) family distribution. Looking durations, approach latencies and distance changes were log transformed (log(x + 1)). Significant *p*-values of the predictor variables at the 5% level are bolded and trends at the 8% level are underlined. R^2^ values were: 0.38 for model A, 0.27 for model B, 0.38 for model C, 0.13 and for model D.

### Effects of association partners on reactions to the experiment apparatus

In 93 of the 170 trials, association partners who approached the apparatus were present. Their presence had a significant positive effect on focal individuals’ looking durations at the experiment apparatus (Table 1 – model A, Fig. 3A). There was no statistically significant effect on the presence of association partners who approached the apparatus on the probability that the focal touched the apparatus (Table 1—model E). However, in 11 of the 14 trials in which individuals touched the apparatus, an association partner who approached the apparatus was present (Fig. 3E). Association partners who approached the apparatus had no effect on their latencies to approach the apparatus (Table 1—model B, Fig. 3B). Furthermore, individuals approached significantly closer to the experiment apparatus (measured by distance change towards the apparatus controlled for the initial exposure distance) when there was a party member present who also approached the apparatus (Table 1—model C, Fig. 3C). The probability that the focal individual showed signs of agitation was not affected by the presence of party members who approached the apparatus (Table 1—model D, Fig. 3D).

### Effects of food availability on reactions to the experiment apparatus

Habitat food availability had a significant positive effect on looking durations at the experiment apparatus (Table 1—model A, Fig. 4A). There was no statistically significant effect of habitat food availability on the probability that the focal individuals touched the experiment apparatus (Table 1—model E, Fig. 4E). There was a positive effect of food availability on latencies to approach the experiment apparatus (Table 1—model B, Fig. 4B). Habitat food availability had no effect on how closely individuals approached the experiment apparatus (measured by distance change towards the apparatus controlled for initial exposure distance, Table 1—model C, Fig. 4C), and no effect on the probability that the focal individual would show signs of agitation during the experimental trial (Table 1—model D, Fig. 4D).

## Discussion

This study aimed to investigate how intrinsic and extrinsic factors affect curiosity, i.e., novelty responses and exploratory tendencies in wild orangutans. In contrast to classic studies on novelty reactions, we relied on a naturalistic design using natural materials. Our results showed that age, the presence of association partners that approached the apparatus, and food availability significantly affected levels of neophobia, neophilia, and visual exploration shown towards a novel experiment apparatus. However, overall, individuals were reluctant to directly interact with the apparatus.

Overall, we found that the focal orangutans showed interest in the experiment apparatus as in most trials they visually explored (i.e., looked at) the apparatus (68% of all trials) and approached the apparatus (64% of all trials). In the trials in which visual exploration occurred, individuals spent on average 12% (range: 0.2–88.2) of the trial duration watching which corresponds to an average watching duration of 3 min (range: 2–900 s). In 24% of all trials the focal orangutan showed signs of agitation, which speaks against high levels of neophobia towards the apparatus. In 88% of all trials in which visual exploration occurred and 69% of all trials in which agitation occurred, it started within the first minute of the beginning of the trial (range 0–9 min for agitation and, suggesting that these reactions were primarily directed at the experiment apparatus. In 64% of all trials, the individuals approached the experiment and in 66% of these cases to 0–5 m. However, in only 8% of all trials, individuals touched the apparatus which suggests that they were hesitant to interact with the apparatus. In 43% of the cases in which individuals touched the apparatus, the touch was indirect via a tool. Tool use allows for investigating objects without physically touching them and thus reduces potential risks of injury that novel objects may present. The fact that the orangutans in our study used tools to interact with the experiment apparatus suggests that the focal individuals perceived the apparatus as a novel object and underlines their reluctance to physically interact with it.

We found that immatures spent significantly more time visually exploring the experiment apparatus than adults (Fig. 1A, Table 1) and showed higher levels of neophilia (i.e., significantly shorter approach latencies and a trend for approaches to closer distances) than adults. This is in line with findings on exploratory tendency and neophilia from a variety of species tested in captivity and the wild, including primates, bats, dogs, hyaenas, and several bird species^9,25,51,63,84–91^; but see^30,45,92^). Furthermore, in wild African great apes, immatures show stronger looking reactions to novel camera trap devices^54^ and are more likely to explore novel food items than other age classes^55^. On the proximate level, higher exploratory tendencies and neophilia in immatures can be explained by the Free-Time Hypothesis (also referred to as Spare-Time Hypothesis), which posits that youngsters have more free time available because they experience reduced environmental stress (such as the need to find food or to be vigilant, which are usually taken care of by their caregivers and/or other group members) and social distractions (such as the ones resulting from mating and reproduction)^14,30,35,36,93,94^. However, in our study, 5 of the 10 immature focal individuals were independently ranging juveniles tested without their mothers. These independent juveniles need to sustain their growing bodies. At the same time, they range largely on their own or in small playful peer groups, and thus likely experience increased environmental stress and social distractions. Therefore, for juveniles, risks of exploration are particularly pronounced^95^. However, from an evolutionary perspective, it stands to reason that immatures have an innate disposition to be exploratory and neophilic because they need to learn about their environment to develop their skill and knowledge repertoires [Needing to Learn Hypothesis:^96^]. For young individuals, the whole world is novel, and thus having temporary mechanisms in place that ensure they can learn about the world is certainly adaptive^13,97^. Higher exploratory and neophilic tendencies in immatures enable them to learn about resources and thus to sustain themselves, which is also in line with the Necessity Hypothesis, which sees ecological needs as the biggest drivers of innovation (see below)^1,4,89,94^.

Interestingly and against our initial prediction, our findings showed that wild immature orangutans have a stronger neophobic reaction to the experiment apparatus than adults in that they were more likely to show signs of agitation during the experimental trials. If high levels of exploration and neophilia in immatures ensure that learning opportunities are realized, high levels of neophobia may protect them from potential dangers while doing so^4,37,45^. A certain level of agitation/excitement and alertness when exploring novel stimuli may minimize risks of predation and injury. At the same time, the heightened/excitement-induced awareness may also improve memory retention and ultimately increase connection formation and therefore learning [reviewed by^98^]. According to the Dangerous Niche Hypothesis, species and individuals which are exposed to higher risks should show higher levels of neophobia^4^. The combination of small body size and semi-solitary lifestyle likely puts immature orangutans at increased ecological risk. However, from our results, it remains unclear if immature orangutans have an intrinsic tendency to be more neophobic than adults or if their neophobic reactions were triggered by them confronting the stimulus more closely (see above). Furthermore, we assessed neophobia via scratching and vocalizations generally associated with fear and agitation^65–67^ . However, scratching and several of the vocalizations that we used are exhibited in several contexts by wild orangutans and are thus not necessarily a definitive indicator of neophobia. High levels of neophobia in combination with high levels of neophilia and exploration have been suggested to be a great ape characteristic^37^. They have also been found in several bird species, including corvids and psittacines which are among the most innovative bird taxa^4,13,99^.

In terms of the effects of social factors, we found that visual exploration, and to some extent neophilia (shown by approach distances but not by approach latencies), increased when at least one association partner was present that approached the experiment apparatus (Figs. 2A and C, Table 1). Consistent with these results, wild orangutans’ natural exploration behavior is positively affected by associations on the developmental and immediate proximate level^59,60^ and wild chimpanzees are more likely to explore novel objects when they are in larger associations^100^. Increased levels of neophilia through social effects are in line with a large number of findings across a variety of taxa whereby the effects range from social facilitation (i.e., the sheer presence of association partners increases, or in some taxa decreases, neophilia) to response facilitation (i.e., the behavior of the association partners towards the novel stimuli elicits corresponding positive or negative reactions towards novel stimuli) [reviewed by^13,37,101^]. However, in contrast to our findings, African great apes show shorter visual exploration of novel stimuli with an increasing number of current association partners^54^. On the one hand, it may be that different ape species vary in their levels of curiosity and how curiosity is influenced by the presence of association partners, as suggested by the Social Curiosity Hypothesis^34^. On the other hand, this study^54^ of African apes did not take the behavior of the association partners into account. Therefore, the seemingly contrasting results may imply that response-specific facilitation leads to increased visual exploration of novel stimuli but not general social facilitation. The Social Information Hypothesis states that individuals confronted with novel stimuli should rely on social cues to assess if the stimuli are worth it and safe to explore^37,52^. Forss et al.^37^ explain the innovation paradox, i.e., that large innovation repertoires are often found in slowly developing species that show a combination of high levels of neophobia and high exploratory tendency: high levels of innate neophobia can be overcome by social information obtained from experts. Our results suggest that rather than affecting neophobia per se, response facilitation leads to an increased likelihood that individuals approach and visually explore the stimuli.

As to environmental effects, we found that high food availability (and, thus, likely, high energy levels^102–105^) correlates with increased visual exploration of the experiment apparatus. However, we also found evidence for lower levels of neophilia (shown in longer latencies to approach the experiment apparatus) when food availability was high. In the innovation literature, there is an ongoing debate about whether necessity or opportunity is the mother of invention, i.e., whether individuals are more prone to innovate when they experience the ecological pressure to do so (e.g., during food shortages or periods of increased energetic stress), or when they encounter suitable ecological conditions and stimuli (e.g., the resources and materials needed for innovations) and have increased amounts of energy and time available^56,94,106,107^. In line with the Opportunity Hypothesis, and findings of other studies^100^, our results suggest that high energy levels increase time investment in gaining information about a novel stimulus. However, the adverse effect of food availability on neophilia (measured in approach latencies) may mean that it is during low energy periods when the stimulus is indeed approached, which then allows for subsequent detailed investigation. If an individual’s energetic state affects exploration and neophilia in different directions, novel stimuli are most likely turned into innovation at a certain optimal level of prevailing ecological pressure rather than at its extremes. Notably, for the Suaq Balimbing population, habitat food availability is generally higher than for most other orangutan populations^108^. The experienced food availability over the course of this study ranged from 5.8 to 14.0. For most orangutan populations, the lower part of this range is around or above the yearly maximum. Therefore, with our experiment, we are likely unable to properly assess the effects of food scarcity on wild orangutans’ curiosity. Furthermore, individuals’ hunger levels may change over the course of the day and between days. Detailed data on food intake over and between days may thus explain further variation in our data.

Our findings may have important implications for experimental behavioral testing of animals, including cognitive tests where it can be difficult to differentiate between low-level performance and a lack of motivation to interact with an experiment apparatus. This is particularly true in the wild, where long habituation periods are often impossible. When testing individuals, the first step is to get them to participate in the experiment. This includes overcoming their fear of the testing procedure and ensuring their motivation to interact with it^37^. Our results imply that individuals’ readiness to participate in behavioral experiments is likely affected by their age and social and environmental factors. These factors should thus all be taken into account when conducting behavioral experiments in the wild.

Across studies of novelty responses and exploration, there is not a clear consensus about which behavioral measures quantify exploration versus neophilia versus neophobia^109,110^. We measured exploration via looking durations and the probability with which the individuals touched the experiment apparatus, whereas other studies have used (or discussed) looking duration as a measure of neophobia^63,111^. Extended periods of watching suggest that the individual is trying to gain information about the novel object. Still, it may be that looking durations (especially shorter ones) are indeed an expression of neophobia. Commonly, exploration is assessed via manipulative actions of the stimuli. In our study, we were not able to analyze effects on manipulative actions with the apparatus because only six individuals touched the experiment apparatus in 14 out of 170 trials.

None of the predictors in the touch probability model showed any significant effects, most likely because touching was so rare and non of the unflanged males touched the apparatus. Larger sample sizes, including more cases in which the individuals touched the experiment apparatus, would allow for a more robust assessment of the potential predictors of individuals’ exploratory tendencies. The details of the trials in which focal individuals touched the apparatus are nevertheless interesting: In eight of the 14 trials in which individuals touched the experiment apparatus the focal individuals touched the experiment apparatus with their hands (or other body parts) and in six trials the focal individuals touched the apparatus with a tool (detached sticks, attached branches, and in one case, a liana). Using a tool to explore a novel stimulus may be less risky than direct body contact. In all trials in which direct physical touch occurred, the individuals that touched the apparatus proceeded to manipulate it via various actions, such as poking, hitting, spinning, and lifting. In all of these cases, the individuals ate from the honey.

Overall, the low rates at which individuals touched the apparatus provide further support that, despite being interested in the experiment apparatus (as evidenced by long looking durations and frequent approaches to close distances), wild orangutans are generally reluctant to directly engage with novelty, especially at their first encounter^52^. On the mechanistic, proximate level, this reluctance may be caused by high levels of neophobia and/or low levels of neophilia. However, strikingly, in only 24% of all trials we saw signs of actively expressed neophobia (i.e., signs of agitation). Furthermore, if neophobia levels were high, we would expect the orangutans to quickly move away from the experiment, but instead, the orangutans stayed in the vicinity of the apparatus for an average of 32 min (range: 6–145 min). On the ultimate level, the reluctance touch the apparatus suggests that in orangutans, the benefits of interacting with novel stimuli do not outweigh its risks^37^. Novel objects and situations represent potential risks to wild animals, for example in the form of injuries, poisoning or predation [reviewed by^4,37,112^]. From an evolutionary perspective, environmental risks are more significant for long-lived (and slowly developing) species, which means that we expect higher levels of neophobia in long-lived species such as orangutans^37^. Interestingly, in a study on innovation in wild chimpanzees, individuals were exploring (i.e., observing from a close distance, sniffing and/or touching) artefacts of a novel foraging situation in around 30% of their visits which are higher rates than we found for the orangutans^100^. This comparison suggests that chimpanzees are less reluctant to interact with novelty either because of lower neophbia or higher neophilia and/or exploration, either due to intrinsic or extrinsic effects, such as having more association partners nearby.

However, despite low rates of physical touch, twenty-one (21) of the 23 focal orangutans tested in this study approached the experiment apparatus in one or more trials, contrasting results from a similar study on the same population where novel objects were positioned on platforms in the canopy^52^. The difference in approach tendencies between the studies could be because our experiment apparatus was largely composed of natural materials, whereas the objects in^52^ were made of unfamiliar materials (e.g., colorful plastic). Our use of a novel object that is familiar in its materials but unfamiliar in its design and configuration, rather than an object made of unfamiliar materials, is an important difference between our study and most studies on novelty reactions, and this makes it difficult to directly compare our findings to findings in other species. However, notably, our experimental design is likely more representative of any type of novelty individuals may indeed encounter in their habitat under natural conditions. Therefore, our naturalistic experimental design may be more appropriate to test novelty reactions in an evolutionary and ecologically meaningful way compared to using objects made of unfamiliar materials. Furthermore, in our study, focal orangutans saw the experiment being installed by human experimenters, which likely played a role in attracting their interest^49^. Previous studies suggest that primates that are habituated to human presence are more ready to interact with novel stimuli than unhabituated individuals [Habituation Hypothesis, proposed by^35^]. Therefore, we cannot rule out that wild orangutans who are not habituated to human presence would have reacted differently to the experiment apparatus and procedure. In our study, the human observers moved away several meters from underneath the apparatus after installing it, and notably, the focal individuals approached, looked at, and uttered their vocalizations while facing the apparatus. However, we cannot assess to what extent orangutans’ reactions to the apparatus were influenced by the presence of human observers.

In our statistical models, we controlled for the effects of exposure distance and trial number because we expected that the variation in these variables would affect our response variables. The results of our models confirmed that with increasing exposure distance, approach distances significantly increased, and there were trends for a decrease in agitation probability and an increase in touch probability (figure S1). Thus, installing the apparatus closer to the focal orangutan increased the likelihood that they visually explored and approached it and increased neophobic reactions. Whereas many studies on novelty use one trial only to assess individual’s reactions to novelty, we here used multiple trials which is more representative of novelty encounters in nature. From the perspective of the individual, novelty arguably remains novel as long as it remains unexplored. Trial number had a significant positive effect on approach latencies and a negative effect on the likelihood that individuals would show signs of agitation (figure S2). As has been found in other species [reviewed by^13^], this shows that with increasing exposure to novel stimuli, individuals gradually become habituated to it and are less neophobic; however, in the case of our study also less eager to approach. Habituation over time to novel stimuli paves the way for further exploration of, and learning about, these stimuli and is thus likely an important element of learning through novelty^4^.

## Conclusion

Our results suggest that immature wild orangutans are neophilic and visually explore novel stimuli longer than adults. They are also more neophobic, a combination that likely allows them to learn safely about their environment. Furthermore, response facilitation through conspecifics increases visual exploration and neophilia in wild orangutans, despite their semi-solitary lifestyle. High energy levels lead to increased investment in gaining information about a novel stimulus, but it may be during low energy periods when the stimulus is indeed approached, which then allows for potential subsequent detailed investigation. Overall, the age effects had larger effect sizes on our measures of curiosity than the effects of association partners or food availability. In other words, wild orangutans are most likely to realize learning opportunities presented by novel stimuli when they are young, while the presence of association partners that show a positive reaction to the stimuli and favorable ecological conditions may further increase their readiness to do so. Therefore, over evolutionary time, extended periods of immaturity, opportunities to depend on the knowledge of conspecifics, and favorable ecological conditions are likely to bring about positive responses to novelty and, thus, potentially high levels of innovativeness.

### Supplementary Information

Supplementary Information 1.

Supplementary Information 2.

## Data Availability

All data analyzed during this study are included in the supplementary information files of this article (Table S1).

## References

[1] van Schaik CP (2016). The reluctant innovator: Orangutans and the phylogeny of creativity. Philos. Trans. R. Soc. B Biol. Sci..

[2] Auersperg AM (2011). Flexibility in problem solving and tool use of kea and New Caledonian crows in a multi access box paradigm. PLoS ONE.

[3] Kaufman JC, Kaufman AB (2004). Applying a creativity framework to animal cognition. New Ideas Psychol..

[4] Greenberg RS, Reader S, Laland KN (2003). The role of neophobia and neophilia in the development of innovative behaviour of birds. Animal innovation.

[5] Webster SJ, Lefebvre L (2001). Problem solving and neophobia in a columbiform–passeriform assemblage in Barbados. Anim. Behav..

[6] Griffin AS, Guez D (2014). Innovation and problem solving: A review of common mechanisms. Behav. Proc..

[7] Herrmann E (2011). A comparison of temperament in nonhuman apes and human infants. Dev. Sci..

[8] Damerius LA (2017). Curiosity boosts orang-utan problem-solving ability. Anim. Behav..

[9] Benson-Amram S, Holekamp KE (2012). Innovative problem solving by wild spotted hyenas. Proc. R. Soc. B Biol. Sci..

[10] Caruso DA (1993). Dimensions of quality in infants' exploratory behavior: Relationships to problem-solving ability. Infant Behav. Dev..

[11] Overington SE (2011). Innovative foraging behaviour in birds: What characterizes an innovator?. Behav. Proc..

[12] Gajdon GK, Lichtnegger M, Huber L (2014). What a parrot’s mind adds to play: The urge to produce novelty fosters tool use acquisition in kea. Open J. Anim. Sci..

[13] Greenberg R, Mettke-Hofmann C (2001). Ecological aspects of neophobia and neophilia in birds. Current ornithology.

[14] Reader SM, Laland KN (2003). Animal innovation.

[15] O’Hara M (2021). Wild Goffin’s cockatoos flexibly manufacture and use tool sets. Curr. Biol..

[16] Preiszner B (2017). Problem-solving performance and reproductive success of great tits in urban and forest habitats. Anim. Cogn..

[17] Thornton A, Isden J, Madden JR (2014). Toward wild psychometrics: Linking individual cognitive differences to fitness. Behav. Ecol..

[18] Smith BR, Blumstein DT (2008). Fitness consequences of personality: A meta-analysis. Behav. Ecol..

[19] Sol D, Lefebvre L, Rodríguez-Teijeiro JD (2005). Brain size, innovative propensity and migratory behaviour in temperate Palaearctic birds. Proc. R. Soc. Lond. B.

[20] Wetzel DP (2017). Problem-solving skills are linked to parental care and offspring survival in wild house sparrows. Ethology.

[21] Szabo B (2022). Wild cognition–linking form and function of cognitive abilities within a natural context. Curr. Opin. Behav. Sci..

[22] Isden J (2013). Performance in cognitive and problem-solving tasks in male spotted bowerbirds does not correlate with mating success. Anim. Behav..

[23] Johnson-Ulrich L, Benson-Amram S, Holekamp KE (2019). Fitness consequences of innovation in spotted hyenas. Front. Ecol. Evol..

[24] Corey DT (1978). The determinants of exploration and neophobia. Neurosci. Biobehav. Rev..

[25] Biondi LM, Bó MS, Vassallo AI (2010). Inter-individual and age differences in exploration, neophobia and problem-solving ability in a Neotropical raptor (Milvago chimango). Anim. Cogn..

[26] Berlyne DE (1966). Curiosity and exploration. Science.

[27] Russell P (1973). Relationships between exploratory behaviour and fear: A review. Br. J. Psychol..

[28] Mettke-Hofmann C, Winkler H, Leisler B (2002). The significance of ecological factors for exploration and neophobia in parrots. Ethology.

[29] Visalberghi E, Janson C, Agostini I (2003). Response toward novel foods and novel objects in wild Cebus apella. Int. J. Primatol..

[30] Kendal R, Coe R, Laland K (2005). Age differences in neophilia, exploration, and innovation in family groups of callitrichid monkeys. Am. J. Primatol. Off. J. Am. Soc. Primatol..

[31] Mettke-Hofmann C (2014). Cognitive ecology: Ecological factors, life-styles, and cognition. Wiley Interdiscip. Rev. Cognit. Sci..

[32] Byrne RW (2013). Animal curiosity. Curr. Biol..

[33] Kidd C, Hayden BY (2015). The psychology and neuroscience of curiosity. Neuron.

[34] Forss S, Willems E (2022). The curious case of great ape curiosity and how it is shaped by sociality. Ethology.

[35] Forss SIF (2022). Captivity and habituation to humans raise curiosity in vervet monkeys. Anim. Cogn..

[36] Kummer H, Goodall J (1985). Conditions of innovative behaviour in primates. Philos. Trans. R. Soc. Lond. B Biol. Sci..

[37] Forss SI, Koski SE, van Schaik CP (2017). Explaining the paradox of neophobic explorers: The social information hypothesis. Int. J. Primatol..

[38] Deaner RO, Van Schaik CP, Johnson V (2006). Do some taxa have better domain-general cognition than others? A meta-analysis of nonhuman primate studies. Evol. Psychol..

[39] Reader SM, Hager Y, Laland KN (2011). The evolution of primate general and cultural intelligence. Phil. Trans. R. Soc. B.

[40] ManyPrimates, D. et al., *Collaboration and open science initiatives in primate research*, in *Primate Cognitive Studies*. 2021, Cambridge University Press.

[41] Tomasello M, Call J (1997). Primate Cognition.

[42] Whiten A (1999). Cultures in chimpanzees. Nature.

[43] Robbins MM (2016). Behavioral variation in gorillas: Evidence of potential cultural traits. PLoS ONE.

[44] Van Schaik CP (2003). Orangutan cultures and the evolution of material culture. Science.

[45] Forss SIF (2019). Differences in novel food response between Pongo and Pan. Am. J. Primatol..

[46] Gustafsson E (2014). Food neophobia and social learning opportunities in great apes. Int. J. Primatol..

[47] Ueno A, Matsuzawa T (2005). Response to novel food in infant chimpanzees: Do infants refer to mothers before ingesting food on their own?. Behav. Proc..

[48] Kendal R (2015). Chimpanzees copy dominant and knowledgeable individuals: Implications for cultural diversity. Evol. Hum. Behav..

[49] Damerius LA (2017). Orientation toward humans predicts cognitive performance in orang-utans. Sci. Rep..

[50] Visalberghi E (2002). Responses to novel foods in captive chimpanzees. Zoo Biol. Publ. Aff. Am. Zoo Aquar. Assoc..

[51] Massen JJ (2013). A behavioral view on chimpanzee personality: Exploration tendency, persistence, boldness, and tool-orientation measured with group experiments. Am. J. Primatol..

[52] Forss SI (2015). Contrasting responses to novelty by wild and captive orangutans. Am. J. Primatol..

[53] Benson-Amram S, Weldele ML, Holekamp KE (2013). A comparison of innovative problem-solving abilities between wild and captive spotted hyaenas, Crocuta crocuta. Anim. Behav..

[54] Kalan AK (2019). Novelty response of wild African apes to camera traps. Curr. Biol..

[55] Biro D (2003). Cultural innovation and transmission of tool use in wild chimpanzees: evidence from field experiments. Anim. Cog..

[56] Grund C (2019). Necessity creates opportunities for chimpanzee tool use. Behav. Ecol..

[57] Schuppli, C., et al., The ontogeny of exploratory object manipulation behaviour in wild orangutans*.* Evol. Hum. Sci. **3** (2021). 10.1017/ehs.2021.34 PMC10427332 37588526

[58] Lamon N, Neumann C, Zuberbühler K (2018). Development of object manipulation in wild chimpanzees. Anim. Behav..

[59] Schuppli C (2017). The effects of sociability on exploratory tendency and innovation repertoires in wild Sumatran and Bornean orangutans. Sci. Rep..

[60] Schuppli C (2020). Early sociability fosters later exploratory tendency in wild immature orangutans. Sci. Adv..

[61] Schuppli, C. and C.P. van Schaik, Animal cultures: how we've only seen the tip of the iceberg*.**Evol. Hum. Sci.* **1** (2019). 10.1017/ehs.2019.1 PMC10427297 37588402

[62] Ruff HA, Dubiner K (1987). Stability of individual differences in infants' manipulation and exploration of objects. Percept. Motor Skills.

[63] Bergman TJ, Kitchen DM (2009). Comparing responses to novel objects in wild baboons (*Papio ursinus*) and geladas (*Theropithecus gelada*). Anim. Cogn..

[64] Fragaszy DM, Mason WA (1978). Response to novelty inSaimiri andCallicebus: Influence of social context. Primates.

[65] Pavani S (1991). Factors influencing scratching behaviour in long-tailed macaques (*Macaca fascicularis*). Folia primatol..

[66] Neal SJ, Caine NG (2016). Scratching under positive and negative arousal in common marmosets (*Callithrix jacchus*). Am. J. Primatol..

[67] Hardus, M., A description of the orangutan's vocal and sound repertoire, with a focus on geographical variation*.**Orangutans Geogr. Var. Behav. Ecol. Conser.,* 49–64 (2009).

[68] Sol D (2011). Exploring or avoiding novel food resources? The novelty conflict in an invasive bird. PLoS ONE.

[69] Biondi LM (2020). Variation in boldness and novelty response between rural and urban predatory birds: The *Chimango Caracara*, *Milvago chimango* as study case. Behav. Proc..

[70] Vogel ER (2017). Nutritional ecology of wild Bornean orangutans (Pongo pygmaeus wurmbii) in a peat swamp habitat: Effects of age, sex, and season. Am. J. Primatol..

[71] R Development Core Team, *R: A language and environment for statistical computing*, R Core Team, Editor. 2019, R Foundation for Statistical Computing: Vienna, Austria.

[72] RStudio, T., RStudio: Integrated Development for R. (2020). RStudio.

[73] Bates, D., et al., *Package ‘lme4’.* Linear mixed-effects models using S4 classes. R package version 1.1–5 (2011).

[74] Fox J (2015). Applied regression analysis and generalized linear models.

[75] Dobson AJ, Barnett AG, Chatfield C, Zidek J (2018). An introduction to generalized linear models. Statistical Science Series.

[76] Hothorn, T., et al., Multcomp: simultaneous inference in general parametric models*.* R package version,1.3–2 (2014). 10.1002/bimj.200810425 18481363

[77] Harrell FE (2015). Regression modeling strategies: with applications to linear models, logistic and ordinal regression, and survival analysis.

[78] Hartig, F. and M.F. Hartig, *Package ‘DHARMa’*. (2017).

[79] Jaeger, B., Package ‘r2glmm’*.* R Found Stat Comput Vienna available CRAN R-project org/package= R2glmm. 10.1002/sim, 2017. **3429**.

[80] Jaeger BC (2017). An R 2 statistic for fixed effects in the generalized linear mixed model. J. Appl. Stat..

[81] Wilke, C.O., H. Wickham, and M.C.O. Wilke, *Package ‘cowplot’.* Streamlined Plot Theme and Plot Annotations for ‘ggplot2, (2019).

[82] Wickham, H., *ggplot2: elegant graphics for data analysis*. 2016: springer.

[83] Lüdecke D (2018). ggeffects: Tidy data frames of marginal effects from regression models. J. Open Sour. Softw..

[84] Carter GG (2018). Younger vampire bats (*Desmodus rotundus*) are more likely than adults to explore novel objects. PLoS ONE.

[85] Mayeaux D, Mason WA (1998). Development of responsiveness to novel objects in the titi monkey, Callicebus moloch. Primates.

[86] Miller R, Schwab C, Bugnyar T (2016). Explorative innovators and flexible use of social information in common ravens (*Corvus corax*) and carrion crows (*Corvus corone*). J. Comp. Psychol..

[87] Isler K (2008). Endocranial volumes of primate species: Scaling analyses using a comprehensive and reliable data set. J. Hum. Evol..

[88] O’Hara M (2017). The temporal dependence of exploration on neotic style in birds. Sci. Rep..

[89] Morand-Ferron J (2011). Who are the innovators? A field experiment with 2 passerine species. Behav. Ecol..

[90] Siwak CT, Tapp PD, Milgram NW (2001). Effect of age and level of cognitive function on spontaneous and exploratory behaviors in the beagle dog. Learn. Mem..

[91] Martina C, Cowlishaw G, Carter AJ (2021). Individual differences in task participation in wild chacma baboons. Anim. Behav..

[92] Greggor AL (2020). Age-related patterns of neophobia in an endangered island crow: Implications for conservation and natural history. Anim. Behav..

[93] Laidre ME (2008). Do captive mandrills invent new gestures?. Anim. Cogn..

[94] Reader SM, Laland KN (2001). Primate innovation: Sex, age and social rank differences. Int. J. Primatol..

[95] Janson, C.H., Ecological risk aversion in juvenile primates: Slow and steady wins the race*.* Juvenile Primates: Life history, development, and behavior, (1993).

[96] Ross Caroline, Jones Kate E, Lee PC (1999). Socioecology and the evolution of primate reproductive rates. Comparative Primate Socioecology.

[97] Sherratt TN, Morand-Ferron J (2018). The adaptive significance of age-dependent changes in the tendency of individuals to explore. Anim. Behav..

[98] Mather M (2007). Emotional arousal and memory binding: An object-based framework. Perspect. Psychol. Sci..

[99] Rössler T (2020). Using an innovation arena to compare wild-caught and laboratory Goffin’s cockatoos. Sci. Rep..

[100] Koops K (2022). Field experiments find no evidence that chimpanzee nut cracking can be independently innovated. Nat. Hum. Behav..

[101] Zentall TR (2006). Imitation: Definitions, evidence, and mechanisms. Anim. Cogn..

[102] Knott CD (1998). Changes in orangutan caloric intake, energy balance, and ketones in response to fluctuating fruit availability. Int. J. Primatol..

[103] Harrison ME, Morrogh-Bernard HC, Chivers DJ (2010). Orangutan energetics and the influence of fruit availability in the nonmasting peat-swamp forest of Sabangau, Indonesian Borneo. Int. J. Primatol..

[104] Vogel ER (2012). Bornean orangutans on the brink of protein bankruptcy. Biol. Let..

[105] O’Connell CA (2021). Wild Bornean orangutans experience muscle catabolism during episodes of fruit scarcity. Sci. Rep..

[106] Koops K, Visalberghi E, van Schaik CP (2014). The ecology of primate material culture. Biol. Let..

[107] Fox EA, Sitompul AF, Van Schaik CP (1999). Intelligent tool use in wild Sumatran orangutans. Ment. Gorillas Orangutans.

[108] Wich SA (2011). Forest fruit production is higher on Sumatra than on Borneo. PLoS ONE.

[109] Greggor AL, Thornton A, Clayton NS (2015). Neophobia is not only avoidance: Improving neophobia tests by combining cognition and ecology. Curr. Opin. Behav. Sci..

[110] Takola E (2021). Novelty at second glance: A critical appraisal of the novel object paradigm based on meta-analysis. Anim. Behav..

[111] Lau AR (2021). Titi monkey neophobia and visual abilities allow for fast responses to novel stimuli. Sci. Rep..

[112] Réale D (2007). Integrating animal temperament within ecology and evolution. Biol. Rev..

