# Supplementary material for: Ecological, social, and intrinsic factors affecting wild orangutans’ curiosity, assessed using a field experiment
Source: Sci Rep. 2023 Aug 14;13:13184. doi: 10.1038/s41598-023-39214-2 (PMC10425418; doi:10.1038/s41598-023-39214-2)
Supplement: Supplementary file 1 — Supplementary Information 1. [file 41598_2023_39214_MOESM1_ESM.docx]

**Ecological, social, and intrinsic factors affecting wild orangutans’ curiosity, assessed using a field experiment**

Caroline Schuppli, Lara Nellissen, Luz Carvajal, Alison M Ashbury, Natalie Oliver-Caldwell, Tri Rahmaeti, Isabelle Laumer, Daniel Haun

**Supplementary material**


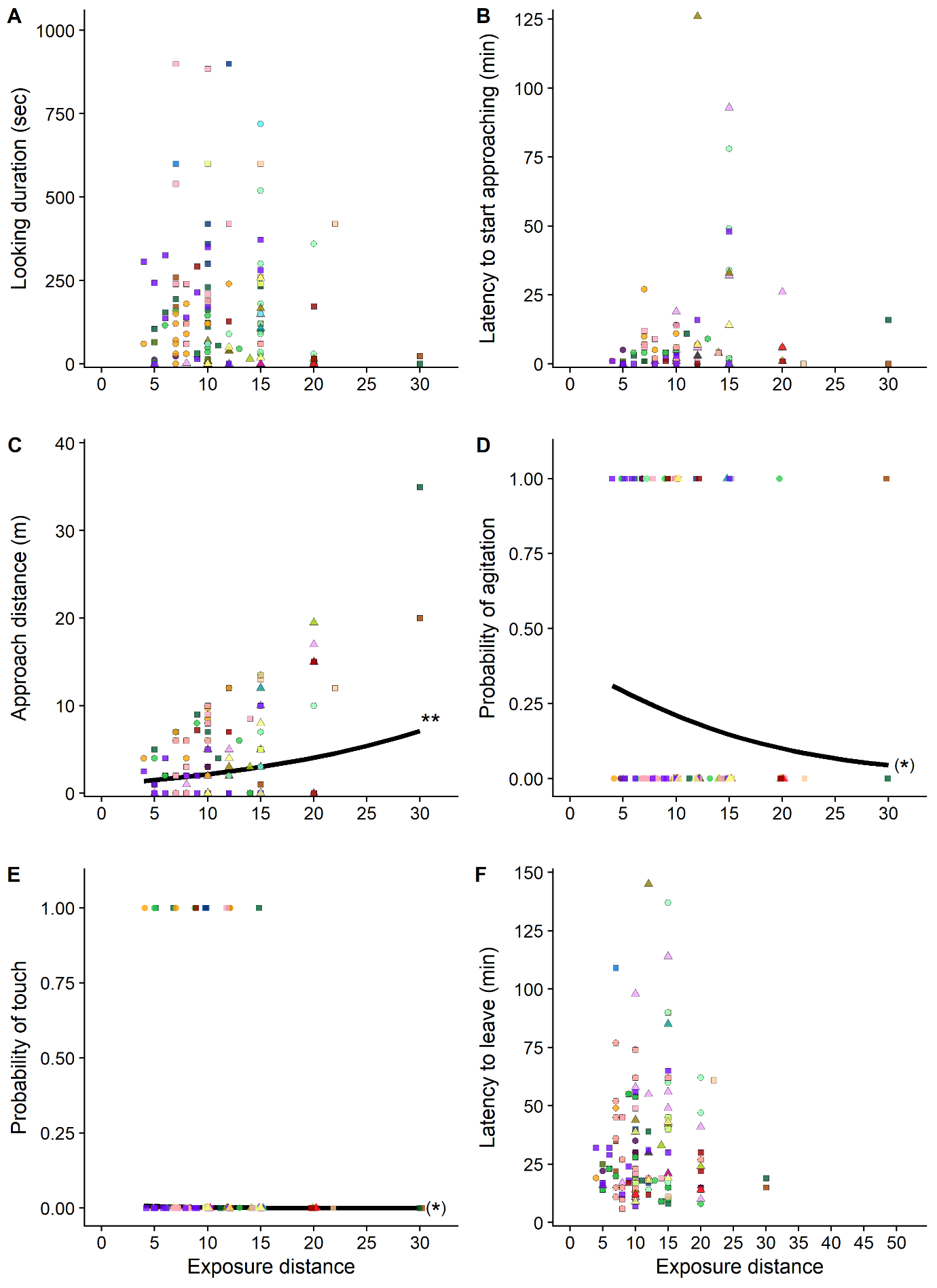


**Figure S1. Effects of exposure distance on reactions to the experiment apparatus.** A) Looking durations at the experimental apparatus, B) latencies to approach the experimental apparatus, C) approach distances towards experimental apparatus, D) the occurrence of signs of agitation during the experimental trial, E) the occurrence of touching the apparatus, and F) the latency to leave the vicinity of the experimental apparatus as a function of exposure distance. For panels A – C and F, each data point represents one experimental trial on one focal individual with colors referring to different individuals, and shape showing their age-sex class (square = immature, circle = mother, triangle = unflanged male). The thick black lines represent mean model predictions across exposure distance values for significant effects, when holding all other predictor variables at their means and the data points with black borders depict the first trial of each individual.


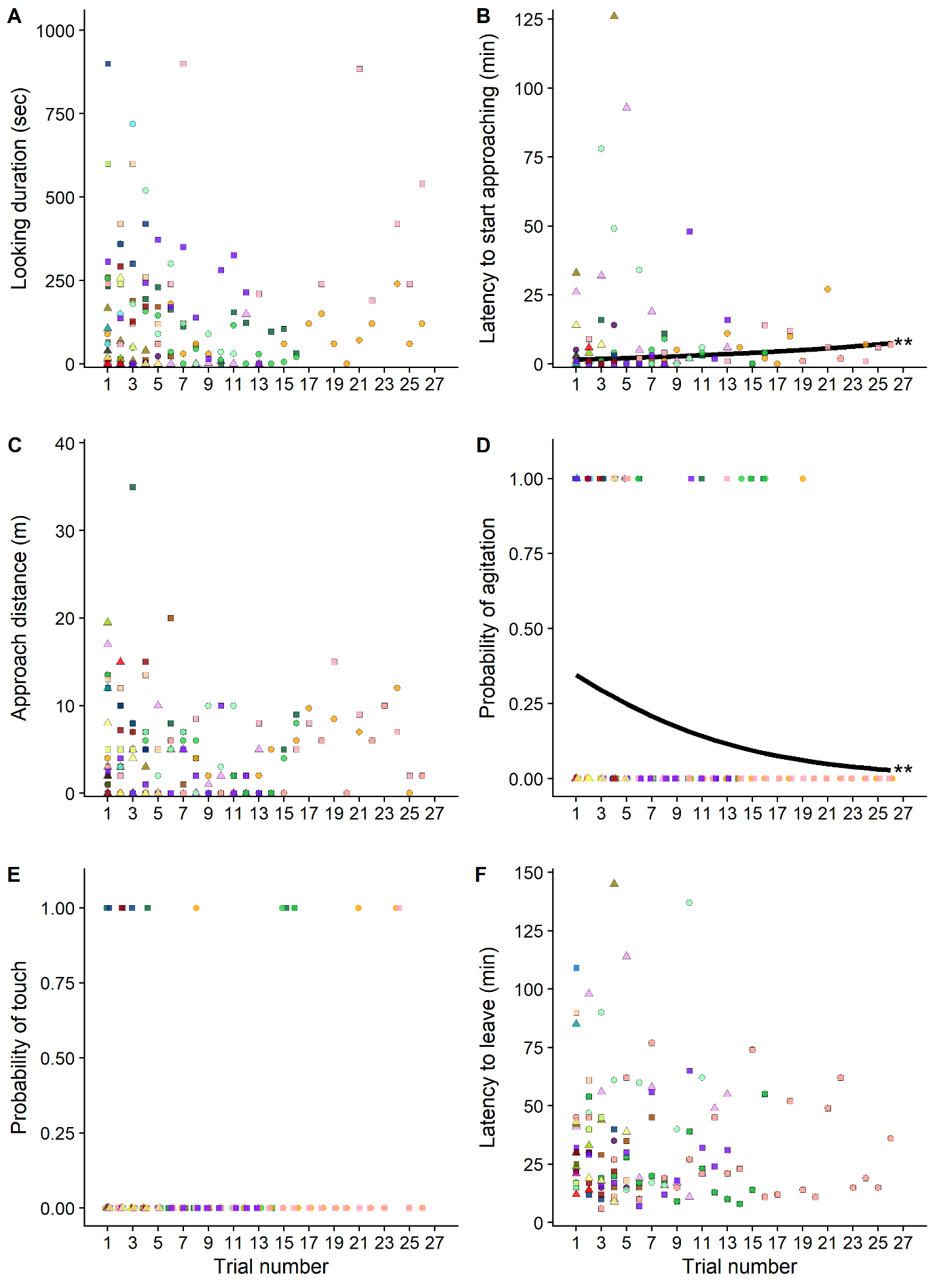


**Figure S2. Effects of trial number on reactions to the experiment apparatus.** A) Looking durations at the experimental apparatus, B) latencies to approach the experimental apparatus, C) approach distances towards experimental apparatus, D) the occurrence of signs of agitation during the experimental trial, E) the occurrence of touching the apparatus, and F) the latency to leave the vicinity of the experimental apparatus as a function of trial number. For panels A – C and F, each data point represents one experimental trial on one focal individual with colors referring to different individuals, and shape showing their age-sex class (square = immature, circle = mother, triangle = unflanged male). The thick black lines represent mean model predictions across trial numbers for significant effects, when holding all other predictor variables at their means and the data points with black borders depict the first trial of each individual.
